# Indicators of depression in elderly and different screening methods

**DOI:** 10.1590/S1679-45082016AO3447

**Published:** 2016

**Authors:** Amanda Gilvani Cordeiro Matias, Marília de Andrade Fonsêca, Maria de Lourdes de Freitas Gomes, Marcos Antonio Almeida Matos

**Affiliations:** 1 Universidade Federal da Bahia, Salvador, BA, Brazil.; 2 Universidade Estadual do Sudoeste da Bahia, Jequié, BA, Brazil.; 3 Escola Bahiana de Medicina e Saúde Pública, Salvador, BA, Brazil.

**Keywords:** Frail elderly, Depressive disorder/diagnosis, Mass screening/methods, Symptom assessment, Questionnaires

## Abstract

**Objective:**

To determine the prevalence of depressive symptoms among elderly and correlate the agreement between the screening methods used.

**Methods:**

A cross-section study of 137 elderly attending the *Programa Vivendo a Terceira Idade* [Living for the Elderly Program]. Depressive symptoms were screened by the Patient Health Questionnaire-9 and the 15-item Geriatric Depression Scale, by Yesavage. Cohen´s kappa analyzed the degree of agreement of these scales.

**Results:**

The prevalence of depressive symptoms screened by the Patient Health Questionnaire-9 was 62.8% and, by the Geriatric Depression Scale, 52.6%. The Spearman correlation between the results of scales obtained rho=0.387, p<0.000. The Kappa reliability coefficient was 0.41 and significance level of p<0.001. The screening methods showed sensitivity of 80% and specificity of 44%.

**Conclusion:**

Both scales showed moderate agreement and were useful for detecting a relevant prevalence of the target outcome of depression among the elderly.

## INTRODUCTION

Psychiatric disorders contribute inexorably to decreased functional capacity and quality of life in the elderly. Among these disorders, depression stands out as a disease with a high frequency worldwide, suggested as the second cause of morbidity for the next decades.^1-3^

Since depressive disease is multifactoral, it may contribute towards greater vulnerability to other morbidities that affect the functional capacity of the elderly. A study analyzed the association between depression and chronic diseases and showed a prevalence of depression of 1.44-fold (95% confidence interval − 95%CI: 1.09-1.92) greater in individuals who reported a chronic disease, and 2.25-fold (95%CI: 1.72-2.94) greater in those with two or more chronic diseases, as compared to persons with no disease.^4^ Other studies pointed out a concern about depressive symptoms with a greater risk for morbidity and mortality, which is more common with aging. They further alert to the fact that people who suffer from depressive disorder may age significantly quicker when compared to those who do not suffer from this condition.^5,6^

Recent research highlights the importance of clarifying the predictive relation between depressive symptoms and functional incapacity.^2,5^ Thus, the strategy of screening common symptoms, which are often ignored as changes in mood, sleep, and appetite, and persist for more than two or three weeks, becomes relevant. These symptoms, despite characterizing the Major Depressive Disorder (MDD), do not make a definitive diagnosis but serve as providential indicators, so as to avoid worse prognoses of the disease.^1,7^

The Diagnostic and Statistical Manual of Mental Disorders, 5^th^ edition (DSM-5)^8^defines MDD as a multidetermined mental health condition characterized by a set of four or more of the following depressive symptoms: changes in mood, appetite, sleep, anhedonia, lethargy, feelings of guilt and low self-esteem, difficulty in concentrating, agitation, and suicidal ideation.

For the diagnosis of depression in an individual, a period of two weeks should be considered, with presentation of at least four of the symptoms listed, including depressed mood or loss of interest or pleasure, or only three more symptoms, if the two cardinal symptoms are present. Mood alterations and anhedonia are cardinal symptoms and at least one should be present for the definitive diagnosis.^2,7^

The National Health Policy for Elderly People addresses preventive action as paramount for assistance guidelines, aiming at an aging process free from disabilities, but it requires effective planning based on a realistic situational diagnosis. These prevention assumptions are relevant for minimizing the incidence of depressive disease in the elderly, since depressive symptoms are associated with their frailty in terms of etiology.^9-11^ Knowledge produced by research may serve as subsidy for evidence-based clinical management, and encourage innovations for the related care programs and policy guidelines.^9,12^

Population health indicators are measured by presence and by absence of disease. Several subjective methods are proposed, such as scales and the validated questionnaires for detection of cases, which should be of good scientific reliability. Considering the difficulties in making diagnosis of depressive disease, due to its subjective and complex nature, some instruments are required. They should be validated and tested with more statistical rigor.

## OBJECTIVE

To determine the prevalence of depressive symptoms among the aged and to correlate the agreement of the screening methods used.

## METHODS

A cross-sectional study was carried out with a population made up of elderly individuals who attend a Center for Social Interaction of the Elderly (CCI), in the city of Vitória da Conquista, State of Bahia (BA), during the period from September to December 2014. The sample of 137 elderly individuals was calculated by the Epi-Info™ application.

Data was collected at the CCI linked to the *ProgramaVivendo a Terceira Idade*, created in 1997, and maintained by the City Administration of Vitória da Conquista (BA). At the time of this investigation, this program had approximately 500 elderly persons enrolled, who participated in weekly activities coordinated by an multidisciplinary team. To prepare the sample of this study, only 324 elderly persons who regularly attended the activities of the program were taken into consideration (minimal acceptable attendance was once every two weeks). To calculate the sample, the prevalence of depression we estimated at 16%, based on a prior study,^13^ adopting an α of 0.05% and a 95%CI, which resulted in a sample of 137 aged people, who were allocated by consecutive convenience.

The inclusion criteria for the research were the elderly attending the CCI, aged ≥60 years, of both sexes, and able to answer the questionnaires. Excluded were those individuals who had cognitive *deficits* as per the evaluation on the Mini Mental State Exam, some difficulty in communication, or were incapable of understanding the data collection instrument.

For the procedure, interviews were used to gather sociodemographic data, in addition to two questionnaires about depressive symptoms. In this study, we used the Patient Health Questionnaire-9 (PHQ-9) and the Geriatric Depression Scale (GDS-15), by Yesavage, which screen depressive symptoms in adults and the elderly who are independent and autonomous.^9,10^

The PHQ-9 evaluates the presence of depressive symptoms according to the DSM-5 protocol, by means of a Likert-type scale composed of nine questions classified in four answer options, that vary from “no, not one day” (zero points) with “almost every day “ (3 points); as a total, the values resulted from zero to 27 points. Hence, the greater the sum of points, the worse the severity of depressive signs.^3,7^ In this study, the cutoff point adopted was ≥9, as recommended.^9^

PHQ-9 is a quick application instrument that screens individuals at greater risk for a major depressive episode. Its screening properties were validated in Brazil for the general population, in 2013.^9^ This scale demonstrated good psychometric and operational properties, with a sensitivity between 77 and 98%, and a specificity of 75 to 80%, and was validated for a population of adults and elderly people.^3,7,9^

The original version of GDS-30 was developed by Sheikh and Yesavage, in the 1980s, and it had 30 items. It was adjusted to the Brazilian population in 1994, as a valid measure for early diagnosis of a depressive episode, according to the criteria of the Diagnostic Manual of Mental Disorders.^14,15^ The GDS-15 is a short version of the original scale, adjusted for the geriatric population by the *Grupo de Estudos de Envelhecimento Cerebral e Demência* [Study Group on Brain Aging and Dementia], and it is available in Portuguese.^14^ Later, this scale was studied for validation of its psychometric properties in Brazil and, currently, it is the second most often used instrument to screen tracking depressive symptoms among the elderly. It is recommended by the World Health Organization.^15-17^

In this study, we used the GDS-15, with 15 affirmative and negative questions, which added up from zero to 15 points, using the cutoff value of ≥6, as per prior studies that applied the same cutoff point, and having the results dichotomized into case/non-case.^10,17^

The descriptive analysis characterized the population studied by frequencies, percentages, means, and standard deviations. Normality of data was verified by the Kolmogorov-Smirnov test and the Q-Q graph. The association of the screened depression signs (PHQ-9 and GDS-15) was checked in two ways: as a dichotomic variable, using Spearman’s test; and as sum of points of each scale, as a numerical variable, through Pearson’s correlation test.

Accuracy (sensitivity and specificity) was calculated between the scales used, with representation of the Receiver Operating Characteristic (ROC) curve. In order to verify the degree of agreement of the scales (methods), Cohen’s kappa test was used with its paired analysis (k<1.0 indicates disagreement and >1.0 indicates complete agreement). We considered α=0.05, 95%CI for all analyses, using the Statistical Package for the Social Sciences (SPSS) software, version 20.0.

The ethical prerogatives were satisfied in the study with human beings, as provided in Resolution 466/12 of the National Health Council and of the National Research Ethics Committee. This study had the prior approval of the Research Ethics Committee of the *Faculdade Independente do Nordeste* (FAINOR), under CAAE number: 33993114.8.0000.5578 and consolidated opinion number 790.750.

## RESULTS

A total of 137 elderly individuals participated in this study, with a mean age of 71.38±7.08 years, in a sample composed primarily of women (65.6%). Most of the elderly were in the 60 to 70 years age range (70.8%). Prevalence of the depressive symptoms screened by the PHQ-9 was 62.8%; the same variable, screened by GDS-15, was 52.6%. The percentages of the sociodemographic data are described on table 1.

**Table 1:** Characteristics of the sample of elderly studied

Variables	n (%)
Age group, years	
60-70	97 (70.8)
71-80	25 (18.8)
>81	15 (10.9)
Gender	
Male	47 (34.4)
Female	90 (65.6)
Marital status	
Living together	71 (51.8)
No partner	66 (42.2)
Depressive signs - GDS-15	
Yes	72 (52.6)
No	65 (47.4)
Depressive signs - PHQ-9	
Yes	86 (62.8)
No	51 (37.2)

GDS-15: Geriatric Depression Scale – 15 items; PHQ-9: Patient Health Questionnaire-9.

**Figure 1:**
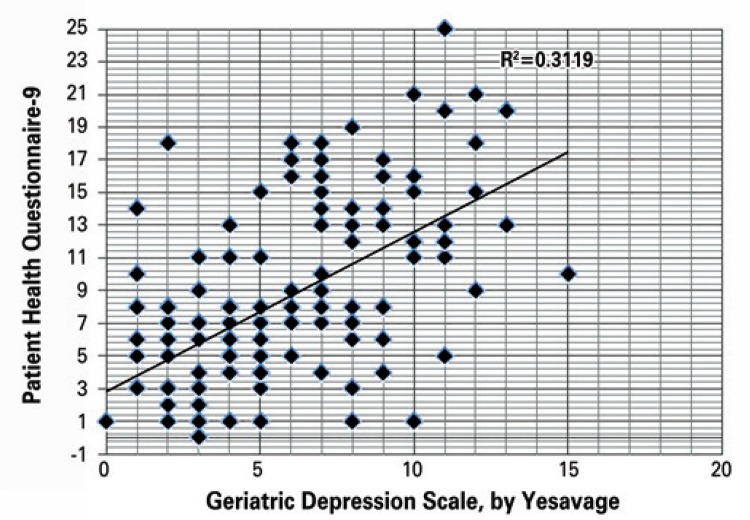
Pearson correlation analysis between the Geriatric Depression Scale and the Patient Health Questionnaire-9, regarding scores indicating depression in the elderly

Spearman’s test was performed among the dichotomized results of the scales and a coefficient rho of 0.387 (p<0.000) was obtained in order to evaluate the association. These scales generated a total score, for which Pearson’s correlation of the sum of the PHQ-9 and the GDS-15 was checked. The coefficient was r=0.56 (p<0.000), and adjusted to *R* ^2^=0.3119, as shown in figure 1.

The aged people screened for the presence of depressive symptoms using the PHQ-9 accounted for 62.8% of the sample; and by the GDS-15, for 52.6%. The difference in detection of the depressive symptoms between these scales was 10.2%. The Cohen’s kappa test obtained the agreement coefficient of 0.40 (p<0.000).

**Figure 2:**
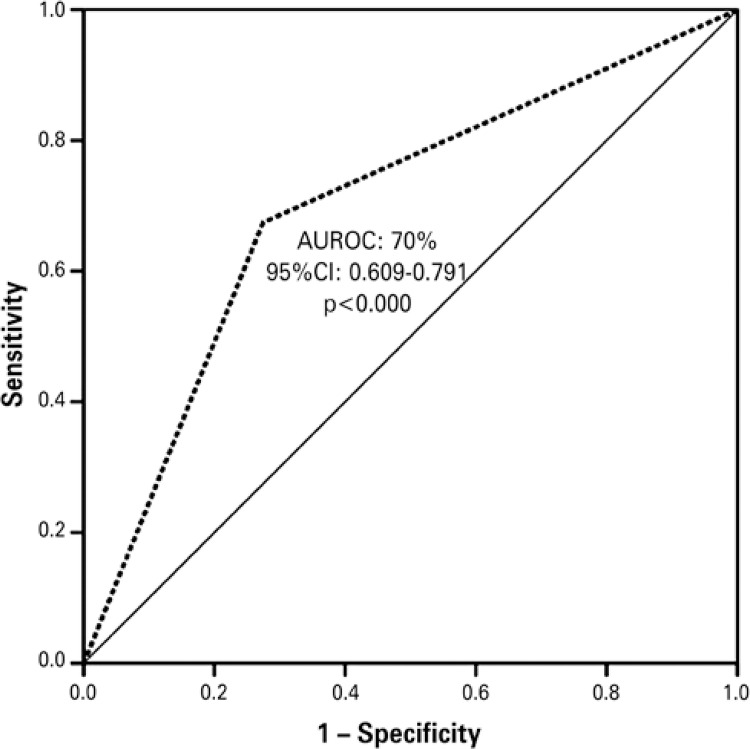
Receiver operating characteristic curves for Geriatric Depression Scale and the Patient Health Questionnaire-9, with cutoff point >6, applied to the sample of elderly individuals AUROC: area under the receiver operating characteristic curve; 95%CI: 95% confidence interval.

The predictive analysis, based on the prevalence of the depressive signs, identified a sensitivity of 80% and specificity of 44%, delimiting the area under the ROC curve of 0.70 (95%CI: 609-791; p<0.000) (Figure 2).

The agreement between the results of GDS and PHQ-9 was statistically significant, according to the intensity measured by the Kappa test (0.42). This agreement is considered moderate.

## DISCUSSION

In this study, composed by a convenience sample among independent elderly individuals, there was a relevant prevalence of the screened depressive symptoms both by PHQ-9 and by GDS. A small mean difference was found, demonstrating acceptable prominence of the validity of the instruments. A possible advantage is still likely to be considered for PHQ-9, due to the lower number of items to be answered, which may make it more acceptable, for its practicality and rapidity. Both scales presented with satisfactory levels of reproducibility of primary outcome evaluated among the elderly. A tendency of greater acceptance may also be observed of the instrument with the fewer number of items.

Another possible explanation on the difference between the prevalence identified by the scales may refer to the cutoff points adopted and the different quantity of items on each scale, since both were analyzed dichotomously. Therefore, due to the supposed overestimation of the PHQ-9 relative to the GDS-15, the kappa test was applied, which demonstrated moderate agreement.

Another presumable explanation for the difference in screened prevalence by the methods would be that the characteristics of these self-reported scales show a subjective response of the individual (as the elderly perceives his/her health and symptoms), or further, the influence of the quantity of five more items in GDS-15 than in PHQ-9. These conjectures, which are useful for early detection of cases, lead to the need for instruments tested and adapted with confirmed screening accuracy.^18^

An analogous study^2^ that applied the GDS-15 found a divergent prevalence of depressive symptoms in the elderly of 18.0%. Another study^17^evaluated 75 elderly people (65-92-year-old) and found a high prevalence of depressive signs (74%) in institutionalized elderly individuals and a smaller difference among non-institutionalized elderly people. These authors report that this scale obtained 84% sensitivity and 95% specificity, but they alert to the fact that it is inappropriate to evaluate individuals with mental *deficit*.^17^

An investigation screened depressive symptoms by the GDS-15, evaluated the presence of depression in 96 elderly people, and identified a significant prevalence of 17.7%, with predominance of females. For decision-making, such symptoms are important aspects to be identified early in the elderly, since depression is surrounded by stigma and prejudice, and emerges with expressive force and concern worldwide.^16^

A study that evaluated the occurrence of depression in primary care settings used the PHQ-9 (cutoff ≥5), with a sample of 4,836 adults and elderly, and identified a relevant general prevalence of 20.1%. It further identified that this disease remains with high indices, but with low therapeutic management, and pointed out that the applicability of the PHQ-9 as a psychometric instrument is useful for this type of active search.^19^

There are still few investigations in Brazil that use the PHQ-9 for screening depressive symptoms, although the instrument has its validity already tested at several healthcare levels, and in several languages and cultural contexts. Only a few studies were carried out with the elderly in the community using this rapid instrument, and that is important to monitor the prevalence of diseases with growing incidence in Brazil and the world, according to an alert by the World Health Organization.^9^

With the objective of evaluating the psychometric properties of the GDS, investigators studied 209 elderly and diagnosed 35.71% of prevalence of depressive disease. They also verified the internal consistency of GDS in 80% relative to The Cambridge Examination for Mental Disorders of the Elderly (CAMDEX) scale. They found an area under the ROC curve of 84% (sensitivity of 79.92% and specificity of 78.29%) and concluded that GDS exhibited reliability, and is useful for screening depressive symptoms.^10^

Additionally, this study tested the accuracy of the scales, and the area under the ROC curve was determined, since it is a test that evaluated the diagnostic and psychometric efficacy of the instrument. GDS-15 and PHQ-9 were correlated, with sensitivity of 80% and specificity of 45% for both applications. Both instruments demonstrated that they are valid tracking measurements of depressive symptoms, with the same cutoff points used in other studies.^3,9,20^

Based on the sensitivity and specificity verified, one can identify a good capacity for identifying positive predictive values (sensitivity of 80% reflects the competence of correctly identifying the outcome), but the capacity for negative predictive identification (specificity of 45% diminishes the ability of the test to be negative in the absence of an outcome of interest) was moderate. The area under the ROC curve of this study was 0.70 (95%CI: 609-791). This index points to satisfactory discriminatory performance of the scales. These prediction scales proved useful and may help in the identification of prevalence of depressive signs, in the clinical environment and in Primary and Secondary Care as an adjuvant, consistent with the recommendation of other researchers.^7,21,22^

The reproducibility studies are important for evaluating methods that require adjustment to subsidize evidence-based practices. A high prevalence of depression detected determines a high degree of agreement expected by chance, and consequently, generates a lower kappa value. The measurement of this test is based on the number of agreement responses and analyzes the reliability of the methods, which is influenced by the prevalence of the study.

In the present study, the Kappa coefficient was 0.41 (95%CI: 0.68-0.90), translating moderate intensity of agreement between the instruments analyzed. The acquiescence of Cohen’s kappa with a 41% coefficient shows moderate adjusted agreement in the screening measurements. Thus, it allows rejecting the hypothesis of equality between the scales used. The interpretation of the Kappa coefficient adopted the Fontelles protocol.^18^

Although the indirect screening methods validated and recommended by the World Health Organization have some advantages (feasible, low cost, and easily applied), they should be tested and confronted in their capacity as epidemiological measurements, aiming to discriminate those of greatest accuracy. This care shows the scientific rigor in evaluating the psychometric capacity of the methods and defines more precise cutoff points, besides elect appropriate scales for specific population groups.

Thus, it is possible to perceive that the literature demands certain questioning in reference to the criteria of designation for the prevalence of depressive disorders screened by the indirect methods; even if validated, it is possible for them to have precision errors. Caution advises the need for robust statistical testing, to decrease error and enhance reliability for the detection of a disease with marked increase in frequency, and estimates of ranking second as most prominent chronic non-communicable disease.^10,12,22^

Based on this discussion, the need for investment in more research of this nature is created to monitor the psycho-emotional aspects with greater regularity, checking reliability of the instruments used and aiming to diminish the margin of errors.

The limitations of this study were reduced sample size, absence of randomization, and possibilities of intrinsic biases of a cross-sectional study, besides being a single-center study. Further investigations are required to pursue realistic knowledge, aiming at elucidative strategies to reduce under-diagnosis. This will lead to not recognizing the disease, hindering decision about its management.

The scales employed proved useful for the detection of depressive symptoms self-reported by independent elderly, and presented a level of agreement with moderate acquiescence for screening such symptoms. The literature searched supports the idea that research on screening is strategically relevant for an active search, seeking the early detection for early preventive decision-making and referrals for diagnostic confirmation.

## CONCLUSION

The scope of the present study evaluated depression suggested as post-modern disease of growing prevalence among the elderly. It also checked the consistency of the Patient Health Questionnaire-9 and of the Geriatric Depression Scale-15 as scales for screening depressive signs. Considering that depressive symptoms are generally underdiagnosed in non-clinical samples, this fact called attention of some researchers, since depression has a relevant impact on function and quality of life of individuals.

By means of this study, it was possible to observe a relevant prevalence of the depressive signs among the elderly, even when considering a non-clinical sample, in which, very often, the onset of depression goes by unnoticed and is underdiagnosed.

We recommend that the subjective screening methods for indications of depression be used as adjuvant to the conventional methods of symptomatic detection, both in the clinical setting and in primary and secondary care, serving as a basis for further confirmation of diagnosis of the disease by a competent physician or professional.

We point out the relevance of good screening practices using simple, low-cost instruments capable of identifying the risks and the dimension of disease as a strategy for consolidation of the preventive and control program of depressive disease, through a system of nurses, physicians, and other professionals active in full healthcare.
