# Supplementary material for: Indicators of depression in elderly and different screening methods
Source: Einstein (Sao Paulo). 2016 Jan-Mar;14(1):6–11. doi: 10.1590/S1679-45082016AO3447 (PMC4872910; doi:10.1590/S1679-45082016AO3447)
Supplement: Translation

# Translation: Indicadores de depressão em idosos e os diferentes métodos de rastreamento

**Authors:** Amanda Gilvani Cordeiro Matias, Marília de Andrade Fonsêca, Maria de Lourdes de Freitas Gomes, Marcos Antonio Almeida Matos

**Affiliations:** 1 Universidade Federal da Bahia, Salvador, BA, Brasil.; 2 Universidade Estadual do Sudoeste da Bahia, Jequié, BA, Brasil.; 3 Escola Bahiana de Medicina e Saúde Pública, Salvador, BA, Brasil.

**Keywords:** Idoso fragilizado, Transtorno depressivo/diagnóstico, Programas de rastreamento/métodos, Avaliação de sintomas, Questionários

## Abstract

**Objetivo:**

Determinar a prevalência dos sintomas depressivos entre idosos e correlacionar a concordância entre os métodos de rastreamentos utilizados.

**Métodos:**

Estudo de corte transversal com 137 idosos vinculados ao Programa Vivendo a Terceira Idade. Os sintomas depressivos foram rastreados pelo *Patient Health Questionnaire*-9 e pela Escala de Depressão Geriátrica de Yesavage-15. O teste Kappa de Cohen analisou o grau de concordância dessas escalas.

**Resultados:**

A prevalência dos sintomas depressivos rastreados pelo *Patient Health Questionnaire*-9 foi 62,8% e, pela Escala de Depressão Geriátrica, foi 52,6%. A correlação de Spearman entre os resultados das escalas obteve rho=0,387, p<0,000. O coeficiente de confiabilidade de Kappa foi 0,41 e significância de p<0,001. Os métodos de rastreamento apresentaram sensibilidade de 80% e especificidade de 44%.

**Conclusão:**

Ambas as escalas apresentaram moderada concordância e foram úteis para detectar uma relevante prevalência do desfecho indicativo de depressão entre os idosos.

## INTRODUÇÃO

Os distúrbios psiquiátricos contribuem inexoravelmente para a redução da capacidade funcional e da qualidade de vida em idosos. Dentre esses distúrbios, a depressão desponta como uma doença de alta frequência mundial, cogitada como a segunda causa de morbidade para as próximas décadas.^1-3^

Visto que a doença depressiva é multifatorial, ela pode contribuir para maior vulnerabilidade a outras morbidades, que comprometem a capacidade funcional do idoso. Estudo que analisou a associação da depressão e doenças crônicas apontou prevalência de depressão de 1,44 (intervalo de confiança de 95% − IC95%: 1,09-1,92) vezes maior em indivíduos que relataram uma doença crônica e 2,25 (IC95%: 1,72-2,94) vezes naqueles com duas ou mais doenças crônicas em relação às pessoas sem doenças.^4^ Outros estudos mostram a preocupação da relação dos sintomas depressivos com maior risco para morbimortalidade mais comum com o avançar da idade. Alertam ainda que pessoas que sofrem de transtorno depressivo podem envelhecer significativamente mais rápido se comparadas àquelas não apresentam esta condição.^5,6^

Recentes pesquisas destacam a importância de esclarecimento sobre a relação preditiva entre os sintomas depressivos e a incapacidade funcional.^2,5^ Assim, torna-se relevante a estratégia de rastreamento de sintomas corriqueiros, que são muitas vezes ignorados, como alteração de humor, do sono e do apetite, e permanecem por mais de duas ou três semanas. Estes sintomas, apesar de caracterizar o Transtorno Depressivo Maior (TDM), não constituem um diagnóstico definitivo, mas servem como indicadores providenciais, a fim de evitarem piores prognósticos da doença.^1,7^

O Manual Diagnóstico Estatístico de Transtornos Mentais 5ª edição (DSM-5)^8^define o TDM como uma condição de saúde mental e multideterminada caracterizado por um conjunto de quatro ou mais dos seguintes sintomas depressivos: alteração do humor, do apetite, do sono, anedonia, letargia, sentimento de culpa e baixa autoestima, dificuldade de concentração, agitação e ideação suicida.

Para o diagnóstico da depressão do indivíduo, deve ser considerado um período de duas semanas, com apresentação de pelo menos quatro sintomas destes listados, entre os quais o humor deprimido ou a perda de interesse ou prazer, ou apenas mais três sintomas, caso os dois sintomas cardinais estejam presentes. Os sintomas de alteração do humor e anedonia são cardinais e pelo menos um deve estar presente para o diagnóstico definitivo.^2,7^

A Política Nacional de Saúde da Pessoa Idosa aborda as ações de prevenção como paradigma primordial para diretrizes assistenciais, visando a um envelhecimento livre de incapacidades, mas requer planejamento efetivo baseado em diagnóstico situacional realístico. Essas prerrogativas de prevenção são relevantes para minimizar a incidência da doença depressiva em idosos, pois os sintomas depressivos aparecem associados às suas fragilidades na base etiológica.^9-11^ O conhecimento produzido pelas pesquisas pode servir de subsídio para o enfretamento clínico baseado em evidências, e incentivar inovações para os programas assistências e diretrizes políticas afins.^9,12^

Indicadores da saúde populacional são medidos pela presença e pela ausência da doença. Vários métodos subjetivos são propostos, como as escalas e os questionários validados para detecção de casos. Estes devem ser de boa confiabilidade científica. Ponderando as dificuldades para o diagnóstico da doença depressiva, por sua subjetividade e complexidade, desponta a necessidade de instrumentos que, além de validados, sejam testados com maior rigor estatístico.

## OBJETIVO

Determinar a prevalência dos sintomas depressivos entre idosos e correlacionar a concordância dos métodos de rastreamentos utilizados.

## MÉTODOS

Foi realizado um estudo de corte transversal com população constituída por idosos vinculados ao um Centro de Convivência do Idoso (CCI), em Vitória da Conquista (BA), no período de setembro a dezembro de 2014. A amostra de 137 idosos foi calculada pelo aplicativo Epi-Info™.

A coleta de dados foi realizada no CCI vinculado ao Programa Vivendo a Terceira Idade, criado em 1997 e mantido pela Prefeitura Municipal de Vitória da Conquista (BA). No momento desta pesquisa, este programa possuía cerca de 500 idosos cadastrados, os quais participavam de atividades semanais coordenadas por uma equipe interdisciplinar. Para compor a amostra deste estudo, foram considerados apenas 324 idosos que frequentavam assiduamente as atividades do programa (a frequência mínima aceitável foi quinzenal). Para base do cálculo amostral, estimou-se uma prevalência de depressão de 16%, baseada em estudo prévio,^13^ sendo admitido α de 0,05% e IC95%, que resultaram em uma amostra de 137 idosos, os quais foram alocados por conveniência consecutiva.

Os critérios de inclusão para pesquisa foram idosos vinculados ao CCI, com ≥60 anos, ambos os sexos e ter capacidade para responder os questionários. Foi excluído o idoso que apresentou *déficits* cognitivos pela avaliação do Mini Exame do Estado Mental, alguma dificuldade de comunicação ou incapacidade de compreender o instrumento de coleta.

Para o procedimento, foram utilizadas entrevistas, para coleta de informações sociodemográficas, e dois questionários sobre os sintomas depressivos. Neste estudo, foi utilizando o *Patient Health Questionnaire*-9 (PHQ-9) e a Escala de Depressão Geriátrica de Yesavage (EDGY-15), que rastreiam a sintomatologia depressiva em adultos e idosos independentes e autônomos.^9,10^

O PHQ-9 avalia a presença de sintomas depressivos, conforme protocolo do DSM-5, por meio de uma escala do tipo Likert composta por nove perguntas categorizadas em quatro opções de respostas, que vão de “não, nenhum dia” (zero pontos) com “quase todos os dias” (3 pontos); no total, os valores resultam de zero a 27 pontos. Assim, quanto maior a pontuação, pior a gravidade dos indícios depressivos.^3,7^ Neste estudo, foi adotado o ponto de corte de ≥9, conforme recomendado.^9^

O PHQ-9 é um instrumento de aplicação rápida, que rastreia indivíduos em maior risco para o episódio depressivo maior. Teve suas propriedades de rastreamento validadas no Brasil para população geral em 2013.^9^ Esta escala tem demonstrado boas características psicométricas e operacionais, com sensibilidade entre 77 e 98% e especificidade de 75 a 80%, tendo sido validada para população de adultos e idosos.^3,7,9^

A versão original da EGDY-30 foi desenvolvida nos anos 1980 por Sheikh e Yesavage, sendo a versão longa com 30 itens. Foi adaptada para população brasileira desde 1994, como uma medida válida para o diagnóstico precoce de episódio depressivo, de acordo com os critérios do manual de diagnóstico de transtornos mentais.^14,15^ A EGDY-15 é uma versão curta da escala original, que foi ajustada para população geriátrica na língua portuguesa pelo Grupo de Estudos de Envelhecimento Cerebral e Demência.^14^ Subsequentemente, essa escala foi objeto de estudo para validação de suas propriedades psicométricas no Brasil e, atualmente, é o segundo instrumento mais empregado para rastrear os sintomas depressivos entre os idosos e recomendada pela Organização Mundial da Saúde.^15-17^

Neste estudo, foi utilizada a EDGY-15, com 15 perguntas afirmativas e negativas, que somam de zero a 15 pontos, tomado o corte de ≥6, de acordo com estudos prévios que adotaram o mesmo ponto de corte e tendo os resultados dicotomizados em caso/não caso.^10,17^

A análise descritiva caracterizou a população estudada pelas frequências, porcentagens, médias e desvios padrão. A normalidade dos dados foi verificada pelo teste Kolmogorov-Smirnov e o gráfico Q-Q. A associação dos indícios de depressão rastreados (PHQ-9 e EDGY-15) foi analisada de duas maneiras: enquanto variável dicotômica utilizou-se o teste de Spearman e, enquanto soma de pontos de cada escala, como variável numérica, utilizou-se a correlação de Pearson.

Foi calculada a acurácia (sensibilidade e especificidade) entre as escalas utilizadas, com representação da curva *Receiver Operating Characteristic* (ROC). Para verificar o grau de concordância das escalas (métodos), foi utilizado o teste Kappa de Cohen, cuja análise pareada (k<1,0 indica discordância e a>1,0 indica concordância completa). Foi considerado α=0,05, IC95%, para todas as análises, utilizando o *SoftwareStatistical Package for the Social Sciences* (SPSS), versão 20.0.

Foram atendidas as prerrogativas da ética na pesquisa com seres humanos, previstas na resolução 466/12 do Conselho Nacional de Saúde e da Comissão Nacional de Ética em Pesquisa. Este estudo teve aprovação prévia pelo Comitê de Ética e Pesquisa da Faculdade Independente do Nordeste (FAINOR) por meio do CAAE: 33993114.8.0000.5578 e parecer consubstanciado 790.750.

## RESULTADOS

Participaram deste estudo 137 idosos com média de idade de 71,38±7,08 anos, em uma amostra composta principalmente por mulheres (65,6%). A maioria dos idosos estava na faixa etária de 60 a 70 anos (70,8%). A prevalência dos sintomas depressivos rastreados pelo PHQ-9 foi de 62,8%; a mesma variável, rastreada pela EGDY-15, foi de 52,6%. Os porcentuais dos dados sociodemográficos são descritos na tabela 1.

**Tabela 1:** Características da amostra de idosos estudados

Variáveis	n (%)
Grupo etário, anos	
60-70	97 (70,8)
71-80	25 (18,8)
>81	15 (10,9)
Gênero	
Masculino	47 (34,4)
Feminino	90 (65,6)
Estado civil	
Em união	71 (51,8)
Sem união	66 (42,2)
Indícios depressivos EDGY-15	
Sim	72 (52,6)
Não	65 (47,4)
Indícios depressivos PHQ-9	
Sim	86 (62,8)
Não	51 (37,2)

EDGY-15: Escala de Depressão Geriátrica de Yesavage; PHQ-9: *Patient Health Questionnaire-9*.

Para avaliar a associação, foi realizado o teste de Spearman entre os resultados dicotomizados dos resultados das escalas e obteve-se coeficiente rho de 0,387 (p<0,000). Estas escalas geraram uma pontuação total, para a qual foi verificada a correlação de Pearson entre a soma do PHQ-9 e da EGDY-15. Foi obtido coeficiente r=0,56 (p<0,000) tendo sido ajustado *R* ^2^=0,3119, conforme figura 1.

**Figura 1:** Análise de correlação de Pearson entre a Escala de Depressão Geriátrica de Yesavage e o *Patient Health Questionnaire-*9, referente à pontuação indicativa de depressão em idosos

Os idosos rastreados com presença de sintomas depressivos utilizando o PHQ-9 somaram 62,8% da amostra, pela EDGY-15, foram 52,6%. A diferença da detecção dos sintomas depressivos entre estas escalas foi de 10,2%. A aquiescência do teste de Kappa de Cohen obteve coeficiente de concordância de 0,40 (p<0,000).

A análise preditiva, partindo da prevalência dos indícios depressivos, identificou uma sensibilidade de 80% e a especificidade de 44%, delineando área sob a curva ROC de 0,70 (IC95%: 609-791; p<0,000) (Figura 2).

**Figura 2:** Curvas *Receiver Operating Characteristic* da Escala de Depressão Geriátrica de Yesavage e do *Patient Health Questionnaire-*9, com ponto de corte >6, aplicadas a amostra de idosos

AUROC: área sob a curva característica de operação do receptor (*area under the receiver operating characteristic*); IC95%: intervalo de confiança de 95%.

A concordância entre os resultados da EDGY e do PHQ-9 foi estatisticamente significativa, de acordo com a intensidade medida pelo teste de Kappa (0,42). Esta concordância é considerada moderada.

## DISCUSSÃO

Neste estudo, constituído por uma amostra de conveniência entre idosos independentes, houve relevante prevalência dos sintomas depressivos rastreados tanto pelo PHQ-9 como pela EDGY. Foi encontrada pequena média de diferença, demonstrando proeminência aceitável de validade dos instrumentos. Considera-se ainda possível vantagem do PHQ-9, pelo menor número de itens a serem respondidos, o que pode torná-lo mais aceito, por sua praticidade e rapidez. Ambas as escalas apresentam níveis satisfatórios de reprodutibilidade do desfecho primário avaliado entre os idosos. Também pode ser observada uma tendência de melhor aceitação ao instrumento com menor número de itens.

Outra possível explicação sobre a diferença entre as prevalências identificadas pelas escalas pode se referir aos pontos de cortes adotados e à quantidade diferente de itens de cada escala, pois ambas foram analisadas de forma dicotômica. Assim, em virtude da suposta superestimação do PHQ-9 em relação à EDGY-15, foi aplicada a testagem de Kappa, que demonstrou concordância moderada.

Outra presumível explicação para diferença da prevalência rastreada pelos métodos seria que as características dessas escalas autorreferidas retratam uma resposta subjetiva do indivíduo (como o idoso percebe sua saúde e sintomas), ou ainda a influência da quantidade de cinco itens a mais na EDGY-15, em relação ao PHQ-9. Essas conjecturas, que são úteis para detecção precoce de casos, remetem à necessidade de instrumentos testados e adaptados com provada acurácia de rastreio.^18^

Estudo^2^ análogo que aplicou a EDGY-15 encontrou divergente prevalência dos sintomas depressivos em idosos de 18,0%. Outro estudo^17^avaliou 75 idosos (65 a 92 anos de idade) e encontrou alta prevalência dos indícios depressivos (74%) em idosos institucionalizados e menor diferença entre idosos não institucionalizados. Estes autores relatam que essa escala obteve sensibilidade de 84% e especificidade de 95%, mas alertaram que é inapropriada para avaliar indivíduos com *déficit* mental.^17^

Pesquisa cujos sintomas depressivos foram rastreados pelo EDGY-15, e que avaliou a presença de depressão em 96 idosos, identificou significativa prevalência de 17,7%, com predomínio no sexo feminino. Tais sintomas são importantes aspectos a serem identificados precocemente em idosos, para a tomada de decisão, visto que a depressão é cercada de estigmas e preconceitos, e emerge com expressiva força e preocupação mundial.^16^

Estudo que avaliou a ocorrência de depressão em ambientes de cuidados primários utilizou o PHQ-9 (corte de ≥5), com uma amostra de 4.836 adultos e idosos, e identificou uma prevalência geral relevante de 20,1%. Constatou ainda que esta doença permanece com índices elevados, mas com baixa abordagem terapêutica, e apontou que a aplicabilidade do PHQ-9 como instrumento psicométrico é útil para este tipo de busca ativa.^19^

Ainda são poucas as pesquisas no Brasil que utilizam o PHQ-9 para rastreamento de sintomas depressivos, embora o instrumento já tenha sua validade testada em diversos níveis de atenção à saúde, e em várias línguas e contextos culturais. Apenas poucos estudos foram realizados com idosos na comunidade, utilizando este instrumento rápido e que tem importância para monitorar a prevalência de doenças com incidência crescente no Brasil e no mundo, conforme tem alertado a Organização Mundial da Saúde.^9^

Com o objetivo de avaliar as propriedades psicométricas da EDGY, pesquisadores estudaram 209 idosos e diagnosticou 35,71% de prevalência da doença depressiva. Eles também verificaram a consistência interna da EDGY em 80% em relação à escala *The Cambridge Examination for Mental Disorders of the Elderly* (CAMDEX). Obtiveram área sob a curva ROC de 84% (sensibilidade de 79,92% e especificidade de 78,29%) e concluíram que a EDGY exibiu boa confiabilidade, sendo útil para o rastreamento dos sintomas depressivos.^10^

Adicionalmente, neste estudo, foi testada a acurácia das escalas, e foi determinada a AUROC, por se tratar de um teste que avalia a eficácia diagnóstica e psicométrica do instrumento. Foram correlacionadas as EDGY-15 e PHQ-9, cuja sensibilidade foi de 80% e a especificidade de 45% para as duas aplicações. Os dois instrumentos demonstraram que são medidas válidas de rastreamento dos sintomas depressivos, com os mesmos pontos de corte utilizados em outras pesquisas.^3,9,20^

Partindo da sensibilidade e da especificidade verificadas, pode-se constatar boa capacidade para identificar valores preditivos positivos (sensibilidade de 80% reflete a competência de identificar corretamente o desfecho), mas a capacidade de identificação preditiva negativa (especificidade de 45% diminui a habilidade do teste de negativa na ausência do desfecho de interesse) foi moderada. A área sob a curva ROC deste estudo foi 0,70 (IC95%: 609-791). Esse índice aponta um desempenho discriminativo satisfatório das escalas. Essas escalas de predição mostraram-se úteis e podem auxiliar na identificação de prevalência dos indícios depressivos, no âmbito clínico e na Atenção Primária e Secundária como adjuvante, compatíveis com as recomendações de outros pesquisadores.^7,21,22^

As pesquisas de reprodutibilidade são importantes para avaliar métodos que necessitam de adequação, para subsidiar práticas baseada em evidências. Uma alta prevalência detectada da depressão determina um alto grau de concordância esperada pelo acaso e, consequentemente, gera menor valor de Kappa. A medida desse teste é baseada no número de respostas concordantes e analisa a confiabilidade dos métodos, que é influenciado pela prevalência do estudo.

No presente estudo, o coeficiente de Kappa foi de 0,41 (IC95%: 0,68-0,90), traduzindo moderada intensidade de concordância entre os instrumentos analisados. A aquiescência do Kappa de Cohen com um coeficiente de 41% retrata concordância ajustada moderada das medições de rastreio. Possibilita, assim, rejeitar a hipótese de igualdade entre as escalas utilizadas. A interpretação do coeficiente de Kappa adotou protocolo de Fontelles.^18^

Apesar dos métodos de rastreamento indiretos, validados e recomendados pela Organização Mundial da Saúde, apresentarem vantagens (factível, de baixo custo e de fácil aplicação), eles devem ser testados e confrontados em sua capacidade enquanto medida epidemiológica, visando discriminar os de melhor acurácia. Este cuidado retrata o rigor científico em avaliar a capacidade psicométrica dos métodos e definir pontos de corte mais precisos, além de eleger escalas apropriadas ao grupo populacional específico.

Assim, é possível perceber que a literatura demanda determinados questionamentos referentes aos critérios de designação para prevalência dos distúrbios depressivos rastreados pelos métodos indiretos; ainda que validados, eles são passíveis de erros de precisão. A cautela advoga a necessidade de testagem estatística robusta, visando à diminuição do erro e ao aumento da confiabilidade, para detecção de uma doença com imponente aumento da frequência e projeções de se tornar a segunda mais proeminente doença crônica não transmissível.^10,12,22^

Com base nesta discussão, desponta a necessidade de investimentos em mais pesquisas desta natureza, para monitorar os aspectos psicoemocionais com maior regularidade, verificando a confiabilidade dos instrumentos utilizados e visando à diminuição da margem de erros.

As limitações deste estudo foram: reduzido tamanho amostral, ausência de randomização e possibilidades dos vieses intrínsecos de um estudo transversal, além de se tratar de pesquisa unicêntrica. Sugere-se a realização de outras pesquisas que busquem conhecimento realístico, visando a estratégias elucidativas para reduzir o subdiagnóstico que implica na ausência do reconhecimento da doença, fato que impossibilita a tomada de medidas terapêuticas.

As escalas utilizadas mostraram-se úteis para detecção de sintomas depressivos autorrelatados por idosos independentes e apresentaram nível de concordância com moderada aquiescência para o rastreio desses sintomas de depressão. A literatura consultada respalda que pesquisas nessa linha de rastreamento são relevantes estrategicamente para busca ativa, visando à detecção precoce para a tomada de decisão preventiva e de encaminhamentos para confirmação do diagnóstico.

## CONCLUSÃO

O escopo do presente estudo avaliou a depressão aventada como doença pós-moderna de crescente prevalência entre idosos. Também verificou a consistência do *Patient Health Questionnaire*-9 e da Escala de Depressão Geriátrica de Yesavage-15 enquanto escalas para o rastreamento dos indícios depressivos. Considerando que os sintomas depressivos geralmente são subdiagnosticados em amostras não clínicas, este fato tem chamado a atenção de alguns pesquisadores, pois a depressão tem relevante impacto sobre a funcionalidade e a qualidade de vida do indivíduo.

Por meio deste estudo, foi possível observar uma relevante prevalência dos indícios depressivos entre os idosos, mesmo se tratando de amostra não clínica, na qual, muitas vezes, o início da depressão passa despercebido e é subdiagnosticada.

Recomenda-se que os métodos subjetivos de rastreio dos indicativos da depressão sejam utilizados como adjuvante aos métodos convencionais de detecção sintomatológica, tanto no âmbito clínico quanto na atenção primária e secundária, servindo de base para posterior confirmação do diagnóstico da doença por um médico ou profissional competente.

Destaca-se a relevância de boas práticas de rastreamento por meio de instrumentos simples, de baixo custo, capazes de identificar os riscos e a dimensão de adoecimento, como estratégia para consolidação de programa preventivo e de controle da doença depressiva, por meio do sistema de enfermagem, médicos e demais profissionais atuantes na atenção integral a saúde.
